# A new gene-scoring method for uncovering novel glaucoma-related genes using non-negative matrix factorization based on RNA-seq data

**DOI:** 10.3389/fgene.2023.1204909

**Published:** 2023-06-12

**Authors:** Xiaoqin Huang, Akhilesh K. Bajpai, Jian Sun, Fuyi Xu, Lu Lu, Siamak Yousefi

**Affiliations:** ^1^Department of Ophthalmology, University of Tennessee Health Science Center, Memphis, TN, United States; ^2^Department of Genetics, Genomics, and Informatics, University of Tennessee Health Science Center, Memphis, TN, United States; ^3^Integrated Data Science Section, Research Technologies Branch, National Institute of Allergy and Infectious Diseases, National Institute of Health (NIH), Bethesda, MD, United States; ^4^ School of Pharmacy, Binzhou Medical University, Yantai, Shandong, China

**Keywords:** glaucoma, RNA-seq, DEG, NMF, BXD strains, pathway analysis

## Abstract

Early diagnosis and treatment of glaucoma are challenging. The discovery of glaucoma biomarkers based on gene expression data could potentially provide new insights for early diagnosis, monitoring, and treatment options of glaucoma. Non-negative Matrix Factorization (NMF) has been widely used in numerous transcriptome data analyses in order to identify subtypes and biomarkers of different diseases; however, its application in glaucoma biomarker discovery has not been previously reported. Our study applied NMF to extract latent representations of RNA-seq data from BXD mouse strains and sorted the genes based on a novel gene scoring method. The enrichment ratio of the glaucoma-reference genes, extracted from multiple relevant resources, was compared using both the classical differentially expressed gene (DEG) analysis and NMF methods. The complete pipeline was validated using an independent RNA-seq dataset. Findings showed our NMF method significantly improved the enrichment detection of glaucoma genes. The application of NMF with the scoring method showed great promise in the identification of marker genes for glaucoma.

## 1 Introduction

Glaucoma is a heterogeneous group of disorders that represents the second leading cause of blindness, affecting up to 91 million individuals worldwide (Quigley, 1996; Goldberg et al., 2000). Glaucoma is characterized by the progressive degeneration of the optic nerve, death of retinal ganglion cells (RGCs), and preferential loss in peripheral visual fields (Jonas et al., 2017). Currently, glaucoma-related expenses are estimated to be $1 billion to $2.5 billion annually. Because glaucoma is an age-related disease, its prevalence is predicted to increase in the coming decades due to the aging population.

Glaucoma diagnosis and treatment response evaluation require a combination of clinical examinations, intraocular pressure (IOP) measurements, and interpretation of visual field and structural imaging parameters. During the early stages of glaucoma, screening techniques based on IOP measurements alone provide low sensitivity, especially in so-called normotensive glaucoma patients whose IOPs are within the normal range (Fernandez-Vega Cueto et al., 2021). Structural signatures such as cup-to-disc ratio likewise provide imperfect sensitivity and specificity for detecting glaucoma (Harper and Reeves, 1999). It is often clinically observed that by the time a glaucoma patient is diagnosed, about 35%–40% of their RGCs (Kerrigan-Baumrind et al., 2000) are lost. Therefore, more sensitive, and specific methods for early diagnosis of glaucoma would be beneficial in order to manage and slow progression, improve treatment response, and ultimately preserve vision (Fernandez-Vega Cueto et al., 2021).

As glaucoma is highly heritable, genetic factors may provide biomarkers for its diagnosis and management and could lead to a better understanding of its pathophysiology (Jonas et al., 2017). Differential gene expression analysis is a commonly used computational approach for identifying marker genes corresponding to a specified phenotype. A typical differential gene expression analysis often identifies a hundred or more differentially expressed genes (DEGs), where a considerable number of them might be highly correlated with one or more other DEGs (Dai et al., 2022).

With the advancement of artificial intelligence, machine learning has provided more accurate glaucoma diagnosis based on imaging, visual field testing, clinical and transcriptomic data using supervised learning; results also have provided biological insight by revealing expression patterns from data using unsupervised learning (Zheng et al., 2019; Alipanahi et al., 2021). Dai et al. (2022) used Logistic Regression (LR), Random Forest (RF), and lasso regression (LASSO) for glaucoma diagnosis based on the DEGs and found diagnosis marker ENO2 by evaluating the efficiency of the classification model and the included features/genes. However, in this study, the classification model was based on a dataset with a very small number of samples (32 samples in total), thus the generalizability of the result is questionable, and needs further validation. Several unsupervised learning methods, such as principal component analysis (PCA) and clustering have been used for discovering patterns from gene expression data for early diagnosis and precise treatment of other diseases (Taguchi and Murakami, 2013; Kallberg et al., 2021; Zhang et al., 2022), but these approaches have several limitations. For example, the value of the PCs cannot represent the gene expression level as it has both positive and negative values. Unsupervised clustering can also be highly sensitive to the metric used to assess similarity, and typically requires subjective evaluation to define clusters.

Non-negative matrix factorization (NMF) is a method based on matrix decomposition, which aims to find two non-negative matrices whose product closely approximates the original matrix. It learns the representation of observations with high dimensionality. As the outcome is non-negative, the algorithm has been applied to a wide variety of problem domains (Lee and Seung, 1999). In recent years, NMF-based methods have been widely applied in genomic data analysis, in particular for identification of cancer-related genes. For example, Brunet et al. (2004) demonstrated NMF is an efficient method for the identification of distinct molecular patterns and provides a powerful method for class discovery based on cancer-related microarray data. They also noted that NMF methods have advantages over other methods, such as hierarchical clustering or self-organizing maps and this was validated based on three different datasets. Kim and Park (2007) applied sparse NMF algorithms to cancer-class discovery and gene expression data analysis. Their results illustrated that the proposed sparse NMF algorithm often achieved a better clustering performance in shorter computing times compared to other existing NMF algorithms. Wang et al. (2013) proposed an NMF based on the maximum correntropy criterion (NMF-MCC) for cancer clustering. They tested the algorithm on six cancer-related gene expression datasets and found the NMF-MCC method was more accurate than other clustering methods. Boccarelli et al. (2018) used NMF to extract biologically relevant genes from gene expression profiles of bone marrow fibroblasts of patients with monoclonal gammopathy of undetermined significance and multiple myeloma. They found those genes may be representative of the considered clinical conditions and may contribute to a deeper understanding of tumor behavior. Zhao et al. (2018) employed the NMF bi-clustering technique to identify subtypes of pancreatic ductal adenocarcinoma, the most widespread form of pancreatic cancer. These studies collectively show that NMF is a powerful tool for pattern and biomarker discovery based on genomic data. Our study is the first to use NMF for identification of glaucoma-related genes. NMF offers distinct advantages for glaucoma-related gene discovery, compared to other options such as the gene scoring approach proposed by Kim and Park (2007), which focused on the contribution of a gene to a cluster. The method used by Kim et al. is more applicable when the goal is to identify subtypes of a disease as all the clusters are of interest; however, it is not ideal for scoring genes within a known, specific target group as is the case for glaucoma genes.

In this study, we used NMF to extract different patterns of gene expression based on eye RNA-seq data of BXD recombinant inbred mouse strains with mutations in *Gpnmb* and *Tyrp1* that are well-known glaucoma-causal genes in mice. Based on our results, we propose a novel gene scoring method which includes the probability of the target group in the corresponding cluster based on the basis matrix of the NMF result. In this case, a higher score represents more importance. As a further test, we applied classical DEG analysis methods and compared results with those obtained using our proposed NMF methods. The enrichment ratios were compared between multiple NMF and DEG outputs using a reliable set of glaucoma-reference genes that were identified from the literature and relevant resources.

## 2 Materials and methods

### 2.1 Dataset

For this study, we generated two sets of RNA-seq data using the Illumina HiSeq 2000 platform (*Mus musculus*). The first dataset consisted of the eye transcriptome from 91 BXD strains aged 12–18 months. Among these, 29 strains had mutations in both *Gpnmb* and *Tyrp1* genes and were treated as the glaucoma group, while 46 strains without mutations in both *Gpnmb* and *Tyrp1* genes were treated as the normal control group. These 75 strains were used as the development and internal test dataset. The remaining 16 strains only have one gene mutation, which could have or have not glaucoma symptoms and they were not included in the analysis. The second dataset consisted of the eye transcriptome from 75 BXD strains aged 2–6 months. Among these, 22 strains had mutations in both *Gpnmb* and *Tyrp1* genes and were treated as the glaucoma group, while 36 strains without mutations were treated as the normal control group. These 58 strains were used as the independent validation dataset. Similarly, the remaining 17 strains only have one gene mutation, and they were not included in the analysis. We considered two different datasets, because we wanted to test our method in two different age groups of mice with the different stage of the same disease carrying mutations in both *Gpnmb* and *Tyrp1* genes and showing glaucoma related phenotypes.

The animals were sacrificed under saturated isoflurane. Eyeballs from the animals were dissected and stored at −80°C until RNA extraction. Total RNA was extracted using Trizol^®^ reagent (Invitrogen, Grand Island, NY, United States) according to the manufacturer’s instructions. Approximately 30 mg of PFC tissue was added into a 2 mL tube containing 700 µL QIAzol Lysis Reagent and one 5 mm stainless steel bead (Qiagen, Hilden, Germany). The tissue was homogenized for 2 min in a Tissue Lyser II (Qiagen, Hilden, Germany) with a speed frequency of 30 r followed by incubation for 5 min. Then, 140 µL chloroform was added into the homogenate, shaken vigorously for 15 s, and centrifuged for 15 min at 12,000 x g at 4°C. Then, 280 µL upper aqueous solution was transferred into a new collection tube containing 500 µL 100% ethanol. The mixture was loaded into a RNeasy mini spin column (Qiagen, Valencia, CA, United States), once with Buffer RWT and twice with Buffer RPE purification. All RNA had been treated with DNase to avoid DNA contamination, and verified by Agilent 2100 Bioanalyzer (Agilent Technologies, Santa Clara, CA, United States). RNA with OD260/280 > 1.8 and RIN >8.0 were used for library preparation. One microgram of RNA was used for cDNA library construction at Novogene using an NEBNext^®^ Ultra RNA Library Prep Kit for Illumina^®^ (cat# E7420S, New England Biolabs, Ipswich, MA, United States) according to the manufacturer’s protocol. Briefly, mRNA was enriched using oligo (dT) beads followed by two rounds of purification and fragmented randomly by adding fragmentation buffer. The first strand cDNA was synthesized using random hexamers primer, after which a custom second-strand synthesis buffer (Illumina, San Diego, CA, United States), dNTPs, RNase H and DNA polymerase I were added to generate the second strand (ds cDNA). After a series of terminal repair, poly-adenylation, and sequencing adaptor ligation, the double-stranded cDNA library was completed following size selection and PCR enrichment. The resulting 250–350 bp insert libraries were quantified using a Qubit 2.0 fluorometer (Thermo Fisher Scientific, Waltham, MA, United States) and quantitative PCR. Size distribution was analyzed using an Agilent 2100 Bioanalyzer (Agilent Technologies, Santa Clara, CA, United States). Qualified libraries were sequenced on an Illumina Novaseq Platform (Illumina, San Diego, CA, United States) using a paired-end 150 run (2 × 150 bases). An average of 40 million raw reads were generated from each library.

The raw reads obtained in fastq format were quality checked using FastQC (https://github.com/s-andrews/FastQC) and then filtered using fastp software to remove reads with adapter contamination, with uncertain nucleotides constituting >10% of either read (N >10%), and with low-quality nucleotides (Base Quality <5) constituting >50% of the read. *Mus musculus* (mouse) reference genome (GRCm38) and gene model annotation files were downloaded from the Ensembl genome browser (https://useast.ensembl.org/). Indices of the reference genome were built using STAR v2.5.0a (Dobin et al., 2013) and paired-end reads were aligned to the reference genome. STAR used the method of Maximal Mappable Prefix which can generate a precise mapping result for junction reads. FeatureCount v0.6.1 (Liao et al., 2014) was used to count the number of reads mapped to each gene. We calculated the Transcripts Per Million (TPM) for each gene in both datasets based on the gene length and the mapped reads. We then rescaled the TPM to log_2_ (TPM+1). Both datasets are available on our GeneNetwork website (www.genenetwork.org) under the names “UTHSC BXD Aged Eye RNA-Seq (Nov20) TPM Log_2_” and “UTHSC BXD Young Adult Eye RNA-Seq (Nov20) TPM Log_2_,” respectively.

### 2.2 Non-negative matrix factorization (NMF) analysis

Given the gene expression matrix *A* (N×M, N-the number of gene, M-the number of samples), *A* was factorized into two matrices with positive entries, *A ∼ WH*. Matrix *W* had size *N×k*, with each of the *k* columns defining a metagene; entry *W*_*ij*_ is the coefficient of gene *i* in metagene *j*. Matrix *H* had size *k×M*, with each of the *M* columns representing the metagene expression pattern of the corresponding sample; entry *H*_*ij*_ represents the expression level of metagene *i* in sample *j*. Given a factorization *A ∼ WH*, we can use matrix *H* to group the *M* samples into *k* clusters. Each sample was placed into a cluster corresponding to the most highly expressed metagene in the sample; that is, sample *j* was placed in cluster *i* if the *H*_*ij*_ was the largest entry in column *j.*

We used the “brunet” method for the NMF with the objective function of KL divergence distance. The method starts by randomly initializing matrices *W* and *H*, which are iteratively updated to minimize a divergence functional.

$$D=\sum_{i,j}A_{i,j}\log\frac{A_{i,j}}{{\left(WH\right)}_{i,j}}-A_{i,j}+{\left(WH\right)}_{i,j}$$

The NMF rank was selected based on cophenetic correlation, dispersion, and silhouette score. The cophenetic coefficient is a metric for robustness of the NMF model and the silhouette score represents the level of separation between clusters, with a value close to 1 indicating dense and well-separated clusters. The stability of the NMF model was evaluated based on consensus matrix. Consensus matrix is an important metric to assess the stability of the clusters obtained for a given rank in NMF modelling. It was computed over multiple independent NMF runs, which is the average of the connectivity matrices of each independent run. Consensus matrix reflects the probability of the sample in the same cluster during the iterations; a value close to 1 representing the model is stable. The NMF model was run 100 iterations at each rank. We used TPM gene expression matrix for NMF modelling.

We constructed the gene score based on the following formula:

$${Gene\_score}_{i}=p*W\left(i,g_{+}\right)+\left(1-p\right)*W\left(i,g_{-}\right)$$ (1)

In this formula, i stands for the *i*th gene, g_+_ is the cluster corresponding to glaucoma, g- is the cluster corresponding to the normal group. P is the precision (the probability of glaucoma samples in cluster g_+_), W (i, g_+_) is the *i*th value of the corresponding cluster of matrix W. The genes were sorted in descending order, based on the gene score calculated using the above formula, with the higher score indicating more importance.

### 2.3 Differentially expressed gene (DEG) analysis

In this study, we used limma-voom from the R package *limma* (Ritchie et al., 2015) to compare the differences in gene expression levels between glaucoma and normal sample groups. We selected differentially expressed genes with different significance thresholds as follows: 1) adjusted *p*-value < 0.05; 2) adjusted *p-*value < 0.05 and |log_2_FC| > 1; 3) adjusted *p*-value < 0.01; 4) adjusted *p-*value < 0.01 and |log_2_FC| > 1; 5) adjusted *p*-value < 0.05 and |log_2_FC| > 0.5; 6) adjusted *p*-value < 0.05 and |log_2_FC| > 1.5; 7) adjusted *p-*value < 0.01 and |log_2_FC| > 0.5; 8) adjusted *p*-value < 0.01 and |log_2_FC| > 1.5.

We used the EnhancedVolcano package (https://github.com/kevinblighe/EnhancedVolcano) to create the volcano plot for DEG results.

### 2.4 Generating a list of known glaucoma-reference genes

The glaucoma-related genes were obtained from multiple publicly available resources using keywords, such as “glaucoma,” “pigmentary dispersion syndrome,” “pigmentary glaucoma,” “ocular hypertension,” “intraocular pressure,” “iris pigment dispersion,” and “corneal calcification.” These keywords were searched in the following databases/repositories: DISEASES database (Pletscher-Frankild et al., 2015) (https://diseases.jensenlab.org/), UniProtKB (UniProt, 2023) (https://www.uniprot.org/uniprotkb), GeneCards (Stelzer et al., 2016) (https://www.genecards.org/), and Alliance database (Alliance of Genome Resources, 2022) (https://www.alliancegenome.org/). Glaucoma-related genes based on manually curated data as well as automatic text-mining were retrieved from the DISEASES database (Pletscher-Frankild et al., 2015). Furthermore, genes with a relevance score ≥3 were also considered from the GeneCards database (Stelzer et al., 2016). The relevance score is calculated by the GeneCards database and is based on term frequency/inverse document frequency (additional details on the scoring can be found here: https://www.genecards.org/Guide/Search#relevance). In brief, the higher the relevance score is, stronger is the association between a gene and term. Finally, a comprehensive list of glaucoma-related genes was derived by combining the above sets and then removing duplicates. This final set obtained is henceforth referred to as the “glaucoma-reference set.”

### 2.5 Functional enrichment analysis of the reference genes

Gene Ontology (GO) and Kyoto Encyclopedia of Genes and Genomes (KEGG) pathway enrichment analyses were performed to further validate the glaucoma-reference set using WebGestalt (Liao et al., 2019).

### 2.6 Comparing NMF and DEG identified genes based on the glaucoma-reference set

The gene lists identified based on NMF and DEG analyses (using *limma*) were compared with the glaucoma-reference genes and the number of overlapping genes was determined. To compare the results across multiple lists and between both methods, we calculated the enrichment ratio based on the following formula:

$$Enrichment\;ratio=\frac{\left(g/n\right)}{\left(G/N\right)}$$ (2)

g - the number of overlapping genes between glaucoma-reference set and NMF/DEGs set.

G - the total number of genes in glaucoma-reference set

n - the number of genes selected from the NMF/DEG approaches.

N - the total number of genes in the mouse genome used in the analysis.

## 3 Results

We used two datasets in this study. We only included the genes which were expressed in at least five samples, resulting in a development and internal testing dataset that included 75 samples (29 glaucoma and 46 normal) and 20,459 genes in the final analysis, and an independent validation dataset that included 58 samples (22 glaucoma and 36 normal) and 19,834 genes for final analysis.

### 3.1 Identifying the optimal NMF rank

We evaluated numerous objective metrics, including cophenetic coefficient, dispersion, explained variance, residuals, and silhouette score. These values were used to identify the optimal rank of the NMF and different ranks were then evaluated, as shown in Supplementary Figure S1. The dispersion and residuals decreased with the increasing rank number, whereas the explained variance increased with increasing rank. The explained variance was consistently higher than 0.90 for all the ranks. Cophenetic correlation coefficient and silhouette scores also indicated that rank for the NMF analysis is 2 (number of clusters).

**FIGURE 1:**
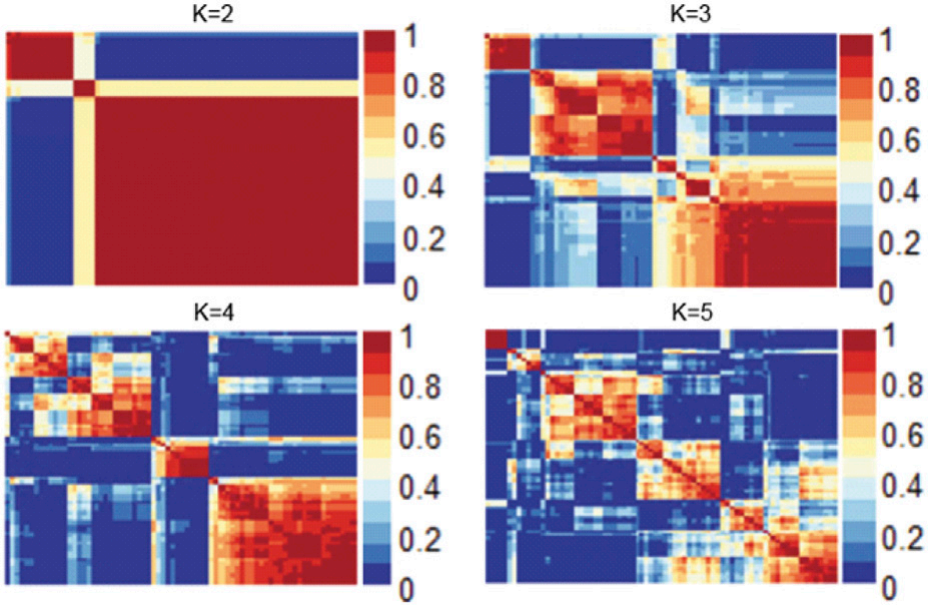
Heatmap of the consensus matrix of the NMF model at ranks ranging from 2 to 5.

Figure 1 shows the consensus matrix of the NMF model at ranks ranging from 2 to 5. As can be seen, the model is stable and consistent when the rank is 2. Collectively, based on the objective metrics, we selected the rank of 2 for further downstream analysis of the gene expression matrix.

### 3.2 Basis and coefficient map

**FIGURE 2:**
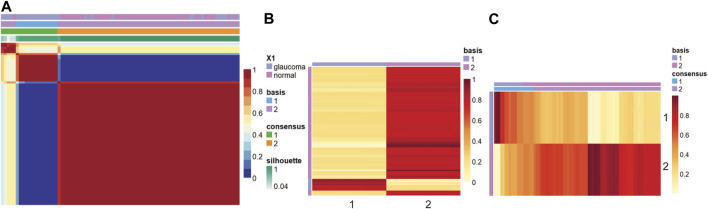
Heatmap of the **(A)** consensus, **(B)** basis, and **(C)** coefficient matrices based on the NMF model with rank 2.

Figure 2 shows the consensus matrix, basis, and mixture coefficients of the NMF model at rank 2, with the label of basis and true label of the strains. Basis coefficient matrix is the matrix W, where rows are the genes, and columns are the metagenes. The mixture coefficient matrix is the matrix H, where rows are metagenes and columns are samples.

In this study, the samples were clustered into two groups with basis 1 and 2. Based on Figure 2A, when comparing the true label with the clustering label, we can label cluster 1 as glaucoma and cluster 2 as normal since all the samples in cluster 1 belong to the glaucoma group, whereas a small fraction of the samples in cluster 2 belong to the glaucoma group. We further characterized clusters and corresponding samples. Cluster 2 included 64 samples (46 normal and 18 glaucoma samples), whereas cluster 1 includes 11 samples (all glaucoma). The precision was calculated as the ratio of true glaucoma samples in cluster 1 to the total number of samples in cluster 1. The gene score was obtained based on formula 1 in the method part. For example, W matrix of gene “Lyz2” is: W1 = 20.64, W2 = 21.20, as cluster 1 was labeled as glaucoma, therefore W _(i, g+)=_20.64, W _(i, g-)_ = 21.20 (i = “Lyz2″), *p* = 11/11 = 1, gene_score_(lyz2)_ = 1*20.64+(1–1)*21.20 = 20.64.

### 3.3 Differentially expressed genes

**FIGURE 3:**
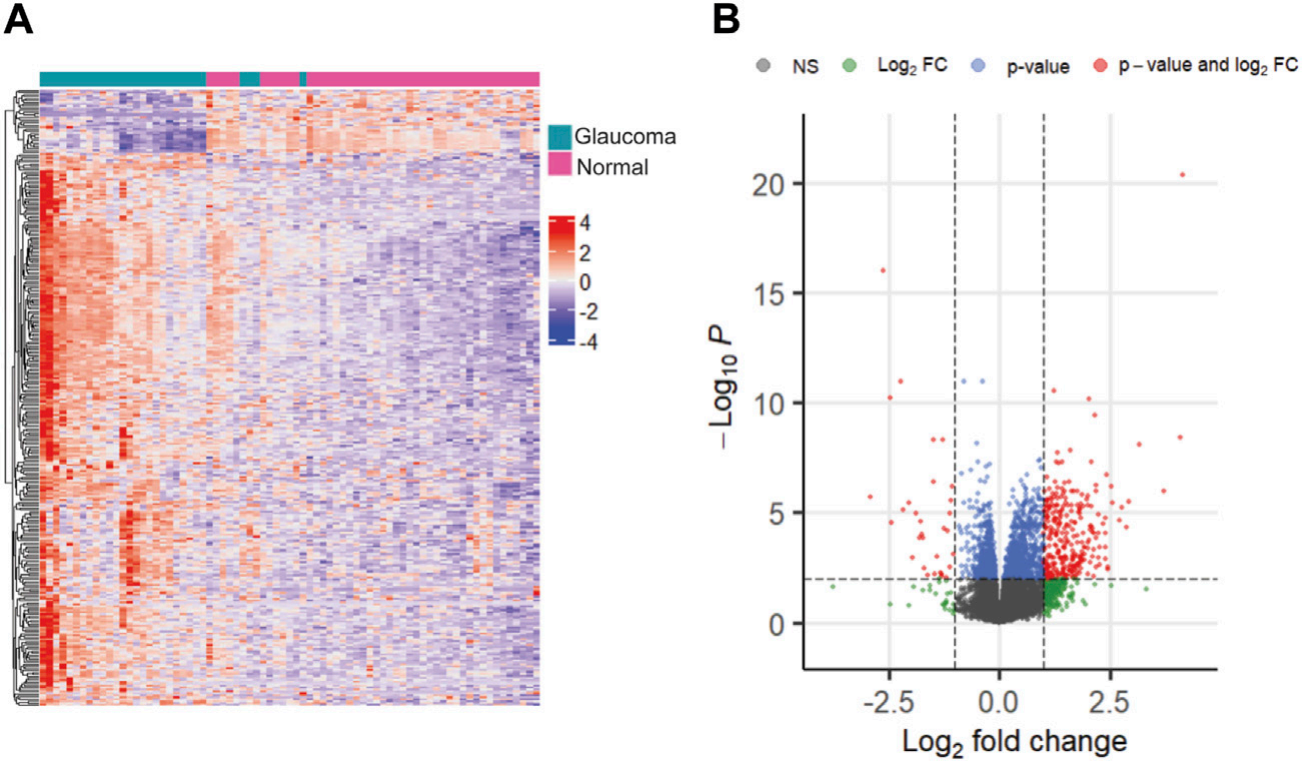
The heatmap **(A)** and volcano plot **(B)** showing differentially expressed genes with adjusted *p-*value < 0.01 and |log2FC| >1.

We selected multiple sets of DEGs based on different significance thresholds. As expected, a threshold of FDR <0.05 resulted in the highest number of DEGs (*n* = 4,088), whereas a threshold of FDR <0.01 & |logFC| >1.5 resulted in the least number of DEGs (*n* = 146), between glaucoma and normal groups. Additionally, we identified 438, 1920, 319, 999, 161, and 700 significant genes at thresholds of FDR <0.05 & |logFC|>1, FDR <0.01, FDR <0.01 & |logFC|>1, FDR <0.05 & |logFC|>0.5, FDR <0.05 & |logFC|>1.5, and FDR <0.01 & |logFC|>0.5, respectively. The representative heatmap and volcano plots of 319 significant DEGs with 286 upregulated and 33 downregulated genes (obtained with a threshold of adjusted *p*-value < 0.01 and |log_2_FC|>1) is shown in Figure 3. Further, the DEG lists obtained with different thresholds were compared with glaucoma-reference set.

### 3.4 Identification of reference list of glaucoma genes and pathway analysis

We searched published literature and other resources and identified 749 genes associated with glaucoma. These reference genes were validated based on KEGG pathway and GO enrichment analyses. The enrichment analysis results clearly indicated the representation of significant pathways and GO annotations of the reference genes. Table 1 shows the top 20 enriched KEGG pathways. Among these, some of the pathways, such as “MAPK signaling”, “PI3K-Akt signaling”, “Ras signaling” have been reported to be associated with glaucoma genes in the literature (Gauthier and Liu, 2017). Supplementary File S1 includes the GO annotations significantly enriched by the glaucoma-reference genes.

**TABLE 1:** Top 20 KEGG pathways significantly enriched by 749 glaucoma reference genes.

KEGG pathway ID	KEGG pathway name	Reference for literature association with glaucoma	FDR corrected *p*-value
**mmu05200**	**Pathways in cancer**	Iglesias et al. (2015)	**0**
mmu04933	AGE-RAGE signaling pathway in diabetic complications	--	0
mmu05418	Fluid shear stress and atherosclerosis	--	1.19E-13
mmu00910	Nitrogen metabolism	--	2.67E-13
mmu05142	Chagas disease	--	5.13E-13
mmu04066	HIF-1 signaling pathway	--	6.56E-11
**mmu05205**	**Proteoglycans in cancer**	Iglesias et al. (2015)	**7.52E-11**
**mmu04010**	**MAPK signaling pathway**	Gauthier and Liu, 2017	**8.5E-11**
mmu05323	Rheumatoid arthritis	--	1.95E-10
**mmu04151**	**PI3K-Akt signaling pathway**	Gauthier and Liu, 2017	**1.16E-09**
mmu05321	Inflammatory bowel disease	--	1.99E-09
mmu04926	Relaxin signaling pathway	--	4.6E-08
**mmu04014**	**Ras signaling pathway**	Gauthier and Liu, 2017	**6.13E-08**
mmu05161	Hepatitis B	--	8.82E-08
mmu05212	Pancreatic cancer	--	1.71E-07
mmu05144	Malaria	--	2.11E-07
mmu00515	Mannose type O-glycan biosynthesis	--	3.98E-07
mmu05210	Colorectal cancer	--	5.16E-07
mmu05225	Hepatocellular carcinoma	--	1.01E-06
**mmu04350**	**TGF-beta signaling pathway**	Iglesias et al. (2015); Gauthier and Liu, 2017	**1.04E-06**

Pathways with known glaucoma associations in literature are highlighted in bold font.

### 3.5 Comparison of genes identified based on NMF and DEG with glaucoma reference genes

The 749 glaucoma reference genes were compared with the DEG-derived genes based on different significance thresholds (Table 2). An equal number of top-ranked NMF-derived genes were considered in order to provide a fair comparison with the glaucoma reference genes. As expected, the DEG- and NMF-derived genes consisting of a higher number of genes, showed better overlap with the glaucoma reference set (Table 2). However, to compare the gene lists within the same method and across different methods, we calculated the enrichment ratio (formula 2) for overlap between the glaucoma-reference set and the NMF- and DEG-derived genes. For example, when using 4,088 number of top genes, there were 198 glaucoma associated genes in DEG result, here *g* = 198, *G* = 749, *n* = 4,088, *N* = 20,459, the enrichment ratio= (198/4,088)/(749/20,459) = 1.32. Our results demonstrated the best enrichment score for DEGs with a threshold of FDR <0.01 & |logFC| >1, whereas the enrichment score was highest when 146 top-ranked genes were considered based on the NMF method. Overall, the enrichment ratio for NMF-derived genes was higher than DEG-derived genes. Interestingly, the enrichment ratio was lowest when maximum number of genes was considered based on both DEG and NMF methods (Table 2). The average enrichment ratio for the overlap was 1.83 and 3.41 across DEG- and NMF-derived genes, respectively (*p* < 0.01, *t-test)*. To be noted, the other genes identified in DEG analysis are not necessarily false positives as these genes could not be validated due to the limited size of the reference gene set.

**TABLE 2:** Comparison of enrichment ratios for glaucoma-reference set between DEG and NMF genes in development and internal testing dataset.

Significance threshold	No. of genes^a^ (DEG)	No. of genes associated with glaucoma (DEG)	Enrichment ratio (DEG)	No. of genes associated with glaucoma (NMF)	Enrichment ratio (NMF)	*p*-value
FDR <0.05	4,088	198	1.32	239	1.60	-
FDR <0.05 & |logFC| >1	438	31	1.93	64	3.99	-
FDR <0.01	1920	111	1.58	151	2.15	-
FDR <0.01 & |logFC| >1	319	27	2.31	51	4.37	-
FDR <0.05 & |logFC| >0.5	999	63	1.72	102	2.79	-
FDR <0.05 & |logFC| >1.5	161	11	1.87	26	4.41	-
FDR <0.01 & |logFC| >0.5	700	53	2.07	84	3.28	-
FDR <0.01 & |logFC| >1.5	146	10	1.87	25	4.68	-
Average enrichment ratio	-	-	1.83	-	3.41	0.006

^a^ Equal number of top-ranked NMF-derived genes were considered for comparison with glaucoma-reference set.

### 3.6 Validation of NMF method using an independent dataset

We validated the NMF method using an independent dataset of glaucoma genes from the BXD mice population. The dataset consisted of 58 BXD mouse strains with 22 glaucoma and 36 normal strains (aged 2–6 months). The results were in agreement with our development and internal test dataset. As shown in Supplementary Figure S2, an NMF rank of 2 resulted in the most robust and stable clusters for this dataset, which is consistent with the original groups with two classes of glaucoma and normal samples; Supplementary Figure S3 shows the consensus, basis, and coefficient maps of the model with rank 2. When looking at the distribution of the true label of strains in the clusters, we found glaucoma samples to be distributed in both clusters 1 and 2. This could be because 2 to 6-month-old mice did not exhibit obvious symptoms of glaucoma. Furthermore, 11 of the glaucoma samples were in cluster 2 (total 26 samples) and 11 were in cluster 1 (total 32 samples). We labeled cluster 2 as glaucoma because a higher percentage of the total samples belonged to the glaucoma group. A representative heatmap and volcano plot for the genes significant with FDR<0.01 & |logFC|>1 is shown in Supplementary Figure S4. Of the 54 DEGs, 50 were upregulated and 4 genes were downregulated in the glaucoma group compared to the normal group. The comparison of the enrichment ratios for the glaucoma reference genes that were in the genes based on the NMF and DEG approaches agreed with the results obtained based on our development and internal testing dataset. The genes identified using the NMF method showed higher enrichment compared to those that were identified using the DEG method (based on the glaucoma-reference genes). In addition, the mean enrichment ratio for the NMF-derived genes was higher and significant (*p* < 0.01) compared to the DEG-derived genes (Table 3).

**TABLE 3:** Validation of the NMF method using an independent dataset.

Significance threshold	No. of genes^a^ (DEG)	No. of glaucoma genes (DEG)	Enrichment ratio (DEG)	No. of glaucoma genes (NMF)	Enrichment ratio (NMF)	*p*-value
FDR<0.05	321	29	2.39	49	4.04	-
FDR<0.05 &|logFC|>1	87	8	2.44	28	8.52	-
FDR<0.01	155	14	2.39	34	5.81	-
FDR<0.01 &|logFC|>1	54	6	2.94	23	11.28	-
FDR0.05 &|logFC|>0.5	166	14	2.23	37	5.90	-
FDR0.05 &|logFC|>FC1.5	32	3	2.48	14	11.59	-
FDR0.01 &|logFC|>0.5	100	10	2.65	29	7.68	-
FDR0.01 &|logFC|>1.5	20	2	2.65	8	10.59	-
Average enrichment ratio	-	-	2.52	-	8.18	0.001

^a^ Equal number of top-ranked NMF-derived genes were considered for comparison with glaucoma-reference set.

## 4 Discussion

In this study, we used NMF to mine RNA-seq data of normal and glaucomatous BXD mice populations in order to identify glaucoma marker genes. Blinded results using the NMF model identified two robust and stable clusters, which were subsequently verified to be consistent with the authenticated labels of the two sample groups. We then developed a novel approach for scoring genes based on the basis matrix of the NMF model and the probability of the glaucoma samples in the corresponding clusters. Our results identified a relatively small number of glaucoma-related genes, based on high enrichment ratio analyses. The NMF method demonstrated significantly improved enrichment ratios for glaucoma-reference genes compared to the traditional DEG analysis. To assure generalizability, we validated the NMF method using an independent dataset and observed similar results. Furthermore, the top 30 genes based on the NMF method applied to the development and internal testing dataset included mt-Co1, mt-Cytb, mt-Nd1, Atp2a1, mt-Nd2, Tnnt3, Lyz2, Myh4, mt-Nd4, mt-Nd6, mt-Nd5, Apoe, Mylpf, Crybb2, Tcap, Cfd, mt-Tp, Eef1a1, Krt5, Krt15, Pvalb, Mb, Aldoa, Tnni2, Ckm, Cryab, Tpm2, Mmp3, Ckmt2, Eno3. Among those, six genes (Lyz2, Apoe, Crybb2, Pvalb, Cryab, Mmp3) were found to be associated with glaucoma when compared with the reference glaucoma genes.

We hypothesize some of these genes are candidate markers for glaucoma. For example, a study reported that mitochondrial lineages that harbor missense mutations in *mt-Co1* may be associated with higher risk of glaucoma in African-American (Collins et al., 2016). Lo Faro et al. (2021) identified an association between POAG and polymorphisms in the mitochondrial genes mt-Nd4 (rs2853496) and mt-Cyb (rs35788393). Atp2a1 encodes one of the Serca Ca (2+)-ATPases, which are intracellular pumps located in the sarcoplasmic or endoplasmic reticula of muscle cells (Odermatt et al., 1996), and Serca1 is also expressed in some non-muscle tissues, including the trabecular meshwork, which is a tissue in the eye that plays a role in regulating intraocular pressure (van Zyl et al., 2020). Thus, Atp2a1 potentially affects the regulation of intraocular pressure and contributes to the development of glaucoma. It has been reported that Tnnt3 plays important roles in the progression of eye disorders, such as glaucoma (Yang et al., 2016). Ckm has been identified as a Myocilin (Myoc) binding partner; binding of mutant Myoc to Ckm changes sarcomere ultrastructure, which may adversely impact muscle function (Lynch et al., 2018). Myoc is the most commonly mutated gene in glaucoma (O'Gorman et al., 2019). Therefore, Ckm may play an important role in glaucoma development. Thus, our results strongly support our hypothesis that NMF can be a promising tool to identify novel genetic markers.

As mentioned earlier, DEG analysis might result in some significant genes which are highly correlated. We investigated this by extracting the top 30 genes from DEG result with the threshold of FDR<0.01 & |logFC|>1 in both datasets and compared with NMF method. About 13% and 32% of the absolute correlation values were higher than 0.75 in the first dataset and second dataset, respectively using DEG, while the ratio was 31% and 14% using NMF using same number of top genes. For the first dataset, the average absolute correlation was not significant between DEG and NMF (mean_DEG_ = 0.52, mean_NMF_ = 0.53, *p* > 0.01, *t*-test), but it was significant in the second dataset (mean_DEG_ = 0.64, mean_NMF_ = 0.37, *p* < 0.01). Therefore, NMF could also get highly correlated genes as it learned representations from all genes, and it could be the case that a group of highly correlated gene jointly contributed to the latent pattern.

The NMF method contains more glaucoma biomarkers than the DEG method might be due to the following: DEG works as a univariate linear model, and it uses hard threshold of both false discovery rate and fold change to decide the significance of the gene. Therefore, DEG method will result in missing some important genes with low fold change. For example, gene “Prph2” (FDR = 0.98, logFC = 0.01), “Rho” (FDR = 0.90, logFC = 0.08), “Cryba2” (FDR = 0.81, logFC = −0.05). However, NMF works as a multivariate model, and it summarizes the contribution from all genes. Since each gene will be weighted by a positive coefficient, NMF is more powerful to capture the important genes with subtle expression changes.

The methods and approach of our novel NMF scoring system are different from the gene scoring approach proposed by others such as Kim and Park (2007) that has been used frequently (Esposito et al., 2020; Wu et al., 2020). When we compared our results obtained using the NMF system with that obtained using methods of Kim and Park (2007), a major difference was seen in the much smaller number of glaucoma-related genes identified by the NMF system. In fact, out of 2,717 significant genes identified from the development and internal testing dataset based on the Kim and Park (2007) approach, only 42 genes were associated with glaucoma (compared against the glaucoma reference genes), with a relatively low enrichment ratio of 0.42. Similarly, when we included 4,305 significant genes identified from the validation dataset, only 79 were associated with glaucoma with a low enrichment ratio of 0.49. One possible reason for this difference might be that the scoring method used by Kim and Park (2007) failed to take into account how well the clusters matched the actual labels and the likelihood of the target group (glaucoma in this particular study) being present in the relevant cluster (i.e., cluster 1 in the development and internal testing set). In Kim’s method, they assigned more weights to the cluster with higher basis value of a gene, which was indicated as p (i, q)*log_2_ (p (i, q). This strategy is more applicable when the purpose is to identify subgroups from the input disease samples and extract potential marker genes for each subtype, which is exactly the intent in those studies (Esposito et al., 2020). In contrast, our study included disease and normal groups, and we were only interested in disease-related genes, so we calculated the gene score based on the precision of the disease group and the basis value and assigned more weights to the cluster corresponding to the disease group. Even with consideration given to differences in overall strategies in our current study and that of Kim and Park (2007), it appears that our NMF approach is more effective (identification of fewer gene candidates with higher enrichment ratios) than the approach of Kim and Park (2007).

Overall, as an unsupervised machine learning model, NMF is good at learning latent pattern from unlabeled data, and it has great potential to discover new target biomarker when reference phenotype is provided, while it needs to be tuned to select the best model. DEG also has its strength as a commonly used method, it is easy to implement, and it can be used for roughly filtering marker genes with hard threshold although it has less power as a univariate model compared to multivariate analysis. Our study also has some limitations. First, the novel gene scoring approach that we developed is more applicable when the dataset includes normal and disease groups instead of subgroups of disease samples. Second, new datasets are warranted to further validate the NMF-derived genes computationally. Finally, the top-rated NMF-derived glaucoma gene candidates will require experimental validation in future studies.

In summary, the current study applied NMF to extract the pattern of gene expression from RNA-seq data collected from normal and glaucomatous BXD strains. The genes were scored based on the basis matrix and the probability of the selected group being present in the corresponding cluster. A list of glaucoma reference genes was derived from literature publications and other sources then validated using KEGG pathway analysis. The enrichment ratio of potential glaucoma markers (based on glaucoma reference genes) were compared between classical DEG and NMF methods. Results showed that NMF identified highly promising candidates and improved the enrichment ratio of the putative glaucoma genes. To evaluate generalizability, the entire study was validated using an independent glaucoma dataset. Our study suggests that NMF is a promising tool for discovering novel marker genes, particularly in glaucoma studies.

## Data Availability

The datasets presented in this study can be found in online repositories. The names of the repository/repositories and accession number(s) can be found below: www.genenetwork.org, GN1022: UTHSC BXD Aged Eye RNA-Seq (Nov20) TPM Log_2_ www.genenetwork.org, GN1021: UTHSC BXD Young Adult Eye RNA-Seq (Nov20) TPM Log_2_.

## Supplementary material

SUPPLEMENTARY TABLE S1 Gene Ontology biological processes enriched by glaucoma reference genes.

SUPPLEMENTARY FIGURE S1 Objective metrics to identify the optical rank (number of clusters) for the NMF analysis. Seven different metrics including Cophenetic—cophenetic coefficient, dispersion—dispersion coefficient, evar—explained variance, rss—Residual Sum of Squares were used to identify the optimal number of clusters.

SUPPLEMENTARY FIGURE S2 Heatmap of the consensus matrix of the NMF model at ranks ranging from 2 to 5 for validation dataset.

SUPPLEMENTARY FIGURE S3 Consensus map **(A)**, basis **(B)** and coefficient map **(C)** of the NMF model at rank 2 for the validation dataset.

SUPPLEMENTARY FIGURE S4 Heatmap **(A)** and volcano plot **(B)** of differentially expressed genes (50 up regulated and 4 down regulated) at adjusted p-value <0.01 and |log2FC|>1 in the validation dataset.
