# Supplementary figures and images for: A new gene-scoring method for uncovering novel glaucoma-related genes using non-negative matrix factorization based on RNA-seq data
Source: Front Genet. 2023 Jun 12;14:1204909. doi: 10.3389/fgene.2023.1204909 (PMC10292752; doi:10.3389/fgene.2023.1204909)

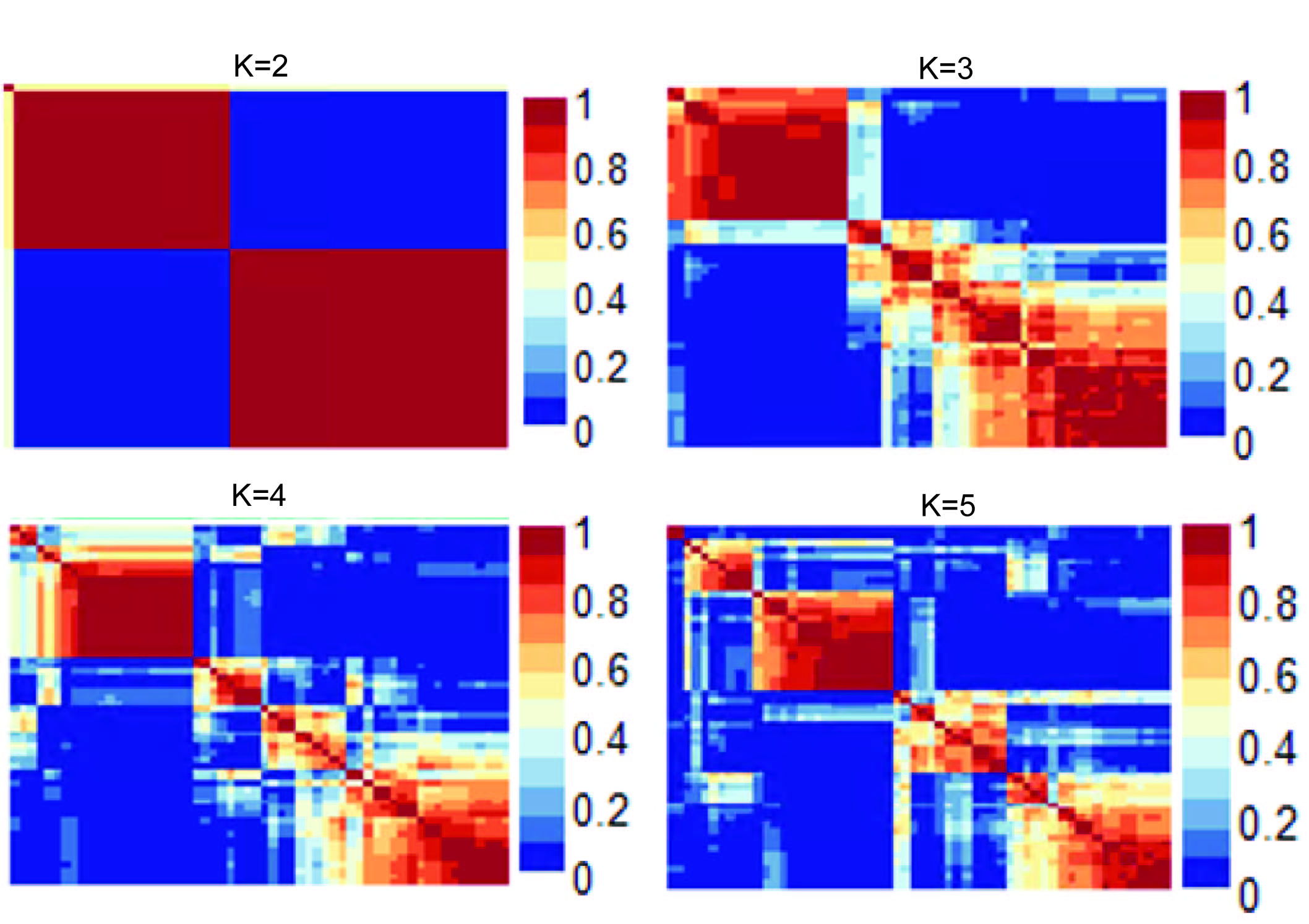

Supplement: Supplementary file 1 [file Image2.jpg]

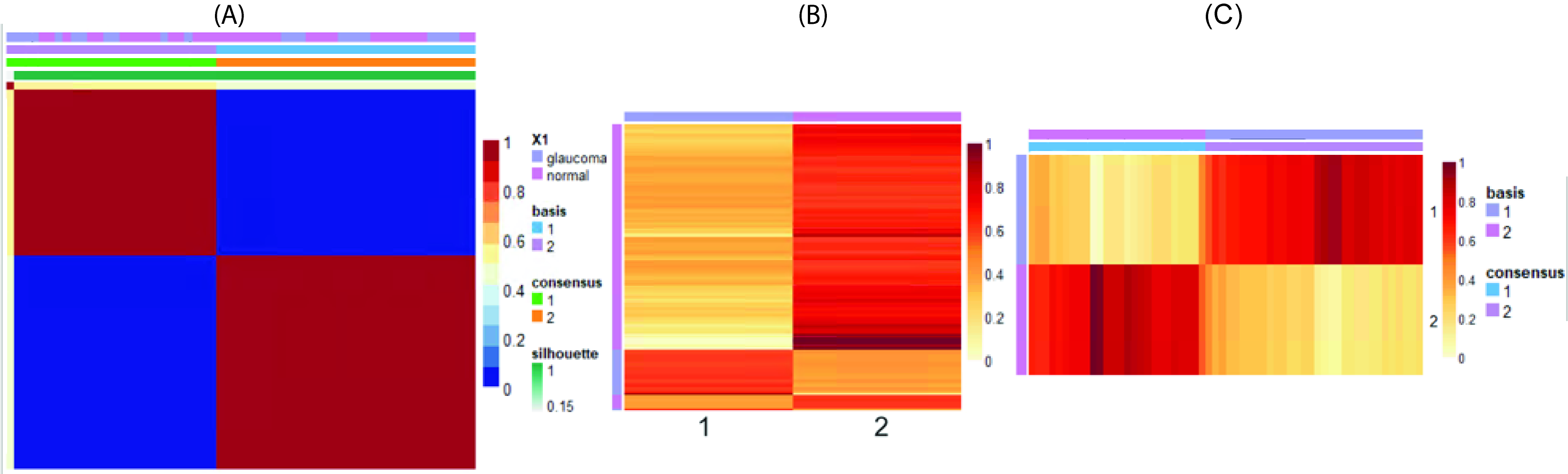

Supplement: Supplementary file 2 [file Image3.TIF]

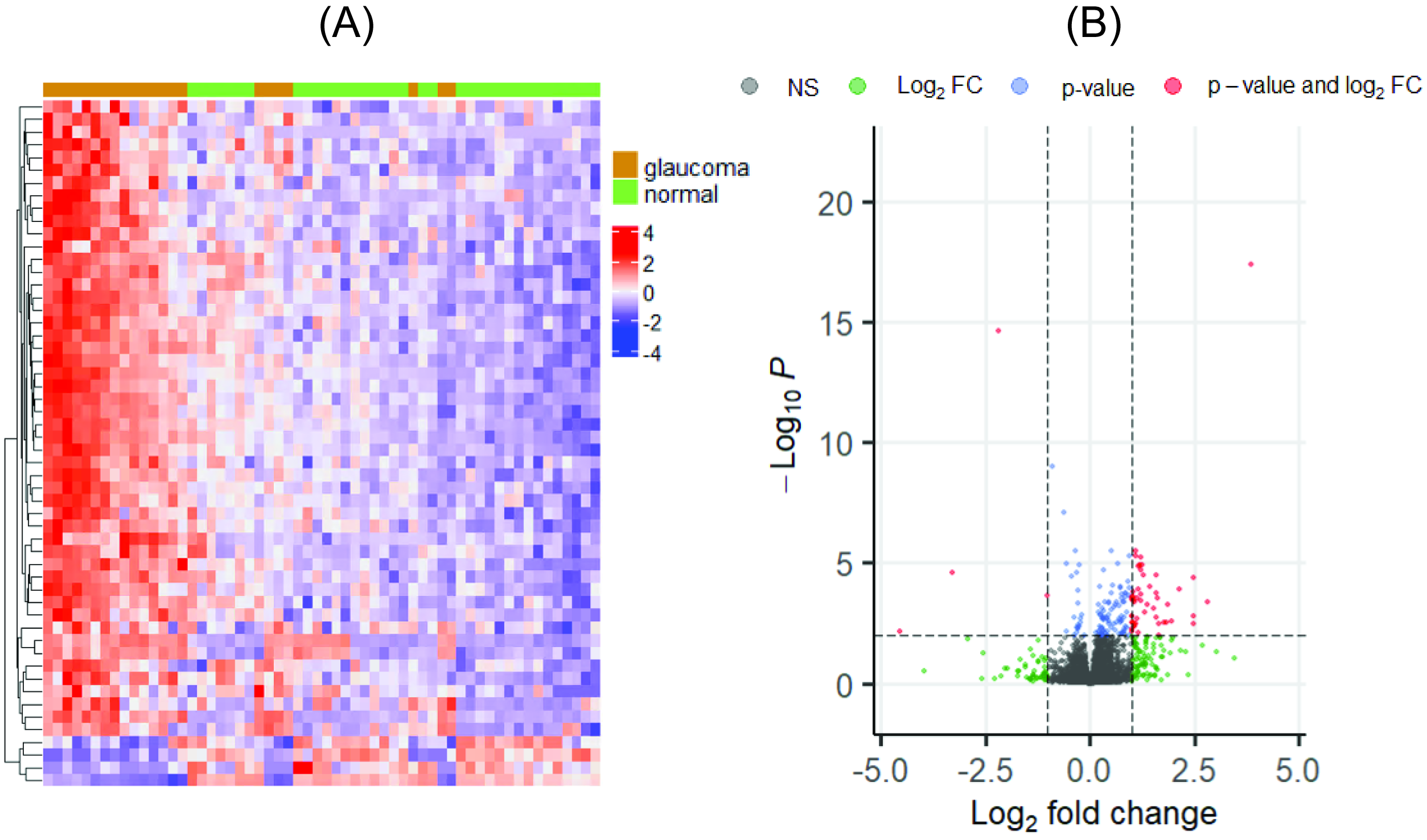

Supplement: Supplementary file 3 [file Image4.TIF]

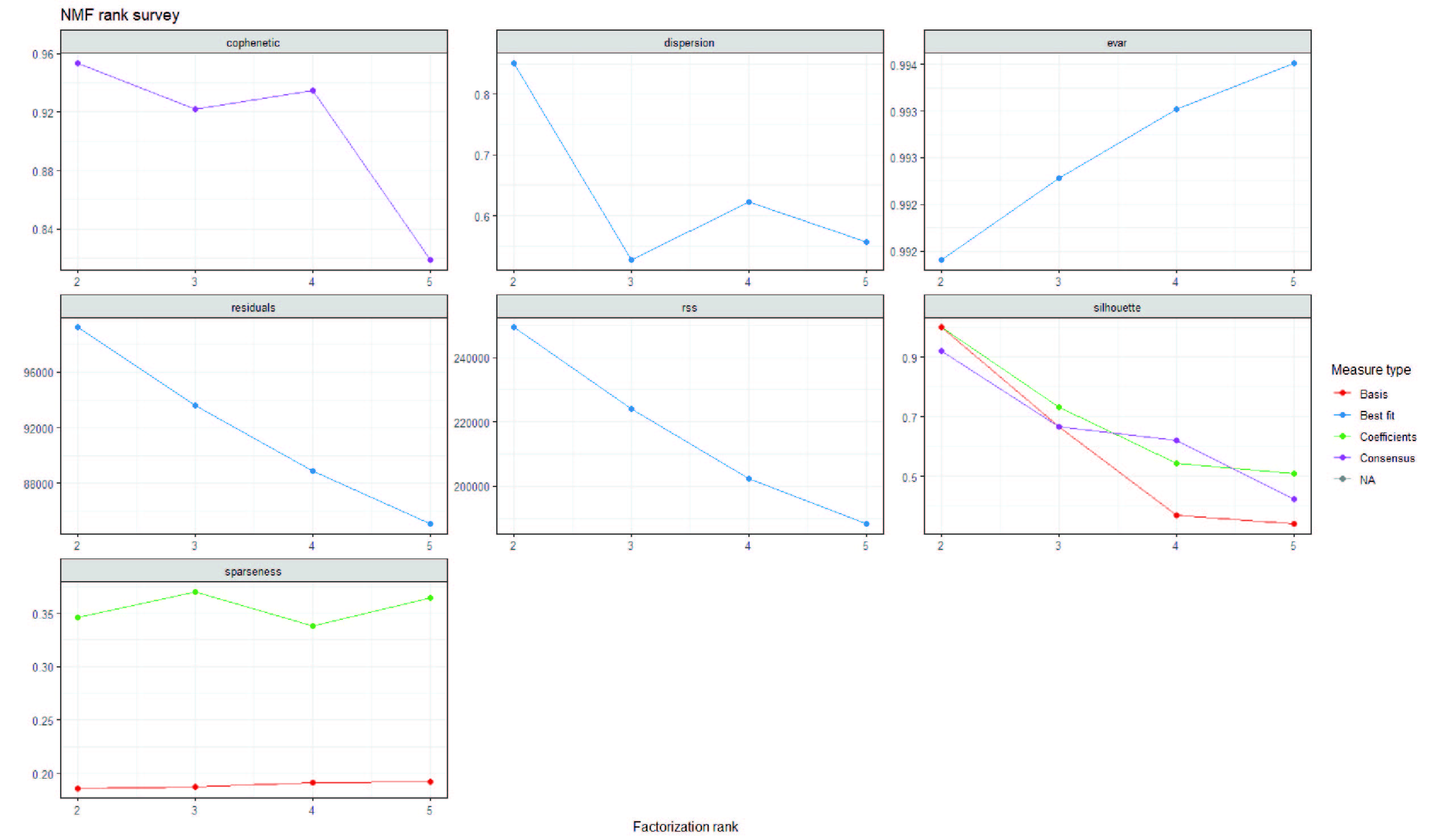

Supplement: Supplementary file 5 [file Image1.jpg]
